# Genome-wide analysis and expression profiling of glyoxalase gene families in oat (*Avena sativa*) indicate their responses to abiotic stress during seed germination

**DOI:** 10.3389/fpls.2023.1215084

**Published:** 2023-06-15

**Authors:** Ming Sun, Shoujiang Sun, Zhicheng Jia, Han Zhang, Chengming Ou, Wen Ma, Juan Wang, Manli Li, Peisheng Mao

**Affiliations:** College of Grassland Science and Technology, China Agricultural University, Beijing, China

**Keywords:** *Avena sativa*, glyoxalase, seed germination, abiotic stress, expression profiling

## Abstract

Abiotic stresses have deleterious effects on seed germination and seedling establishment, leading to significant crop yield losses. Adverse environmental conditions can cause the accumulation of methylglyoxal (MG) within plant cells, which can negatively impact plant growth and development. The glyoxalase system, which consists of the glutathione (GSH)-dependent enzymes glyoxalase I (GLX1) and glyoxalase II (GLX2), as well as the GSH-independent glyoxalase III (GLX3 or DJ-1), plays a crucial role in detoxifying MG. However, genome-wide analysis of glyoxalase genes has not been performed for one of the agricultural important species, oat (*Avena sativa*). This study identified a total of 26 *AsGLX1* genes, including 8 genes encoding Ni^2+^-dependent GLX1s and 2 genes encoding Zn^2+^-dependent GLX1s. Additionally, 14 *AsGLX2* genes were identified, of which 3 genes encoded proteins with both lactamase B and hydroxyacylglutathione hydrolase C-terminal domains and potential catalytic activity, and 15 *AsGLX3* genes encoding proteins containing double DJ-1 domains. The domain architecture of the three gene families strongly correlates with the clades observed in the phylogenetic trees. The *AsGLX1*, *AsGLX2*, and *AsGLX3* genes were evenly distributed in the A, C, and D subgenomes, and gene duplication of *AsGLX1* and *AsGLX3* genes resulted from tandem duplications. Besides the core cis-elements, hormone responsive elements dominated the promoter regions of the glyoxalase genes, and stress responsive elements were also frequently observed. The subcellular localization of glyoxalases was predicted to be primarily in the cytoplasm, chloroplasts, and mitochondria, with a few presents in the nucleus, which is consistent with their tissue-specific expression. The highest expression levels were observed in leaves and seeds, indicating that these genes may play important roles in maintaining leaf function and ensuring seed vigor. Moreover, based on in silico predication and expression pattern analysis, *AsGLX1-7A*, *AsGLX2-5D*, *AsDJ-1-5D*, *AsGLX1-3D2*, and *AsGLX1-2A* were suggested as promising candidate genes for improving stress resistance or seed vigor in oat. Overall, the identification and analysis of the glyoxalase gene families in this study can provide new strategies for improving oat stress resistance and seed vigor.

## 1 Introduction

Reactive dicarbonyl compounds, including methylglyoxal (MG), glyoxal (GO), and 3-deoxyglucosone (3-DG), are the main toxic metabolites spontaneously produced by various metabolic pathways when plants are exposed to stress, and these compounds can hinder normal growth and development (Chinchansure et al., 2015; Ramu et al., 2020). MG, which is the most prevalent reactive dicarbonyl compound, is a by-product of nonenzymatic reactions through glycolysis and the Calvin cycle, and enzymatic pathways through proteins and fatty acid metabolism (Kaur et al., 2014; Takagi et al., 2014). High concentrations of MG can be cytotoxic to cells as they spontaneously form advanced glycation end-products (AGEs) when interacting with nucleic acids, proteins, and lipids (Kaur et al., 2014; Singla-Pareek et al., 2020). However, despite its potential cytotoxicity at high concentrations, MG also serves as a vital signaling molecule involved in various biological processes (Singla-Pareek et al., 2020). Therefore, the maintenance of MG homeostasis is crucial for the normal growth and development of plants.

Plants maintain MG homeostasis through the glyoxalase system and non-glyoxalase system. The former primarily comprises three enzymes, namely, glyoxalase I (GLX1, GLXI or GLYI), also known as lactoylglutathione lyase (EC 4.4.1.5); glyoxalase II (GLX2, GLXII or GLYII), also known as hydroxyacylglutathione hydrolase (EC 3.1.2.6); and glyoxalase III (GLX3, GLYIII or DJ-1)(Li, 2016). GSH spontaneously reacts with MG to form hemithioacetal (HTA), which is then converted to S-D-lactoylglutathione (SLG) by GLX1. GLX2 hydrolyses SLG to produce D-lactate and GSH, which is then recycled back into the system (Thornalley, 1990). The functions of these two GSH-dependent glyoxalases have been extensively explored in plants and reviewed in terms of MG detoxification, cell aging, signal transduction, cell division and differentiation, starch synthesis, pollination, nutrient response, and stress response (Singla-Pareek et al., 2020). Among these, stress response is considered their primary function (Kaur et al., 2014; Hasanuzzaman et al., 2017). The expression and activity of GLX1 and GLX2 vary significantly under various stress conditions, such as hypoxia, salt, drought, heat, cold, and heavy metal (Kaur et al., 2014; Sankaranarayanan et al., 2017). GLX3, a novel glyoxalase enzyme of the DJ-I protein family, directly catalyzes the conversion of MG to D-lactate (Ghosh et al., 2016). Only a few studies have investigated GLX3 in model plants and cereal crops, demonstrating its participation in stress responses (Jana et al., 2021; Kumar et al., 2021; Gambhir et al., 2023). Recent studies have shown that GLX1 and GLX2 are also involved in regulating seed vigor in *Oryza sativa* and *Arabidopsis* (Schmitz et al., 2017; Liu et al., 2022), but their role in seed development, storage, and germination is still poorly understood.

The identification and functional analysis of the glyoxalase gene family members have gained significant attention due to the diverse functions of glyoxalase enzymes. Although *GLX1* and *GLX2* genes have been genome-wide identified in model plants and important crops, such as *Arabidopsis* (Mustafiz et al., 2011), *O. sativa* (Mustafiz et al., 2011), *Sorghum bicolor* (Bhowal et al., 2020), *Glycine max* (Ghosh and Islam, 2016), and *Brassica rapa* (Yan et al., 2018), the genome-wide identification on GLX3 is limited (Li et al., 2019; Jana et al., 2021; Yan et al., 2023). Notably, both *GLX1* and *GLX2* genes in these species have multiple members with diverse subcellular localizations (Li, 2016). GLX1s are classified into two types: Ni^2+^-dependent and Zn^2+^-dependent (Schmitz et al., 2018), while GLX2s belong to the beta-lactamase protein family and have a binuclear metal center consisting of Fe^3+^, Zn^2+^, and Mn^2+^ (Schilling et al., 2003). GLX3s belong to the DJ-1/PfpI superfamily and do not require metal ions for their optimal activity (Ghosh et al., 2016). The identification and analysis of *GLX1*, *GLX2*, and *GLX3* genes are crucial for comprehending the regulation of plant growth, development, and stress response.

Oat (*A. sativa*) is an important cereal and feed crop that has recently had its genome data released (Rasane et al., 2015; Kamal et al., 2022; Peng et al., 2022), but has not yet been extensively studied in terms of the *GLX1*, *GLX2*, and *GLX3* genes. Therefore, the identification and expression analysis of these genes at the whole-genome level will help to rapidly advance stress-resistant breeding and seed vigor improvement in oat. In our previous research, we discovered a close association between the GSH-dependent glyoxalases and the AsA-GSH cycle with oat seed vigor, where GSH content serves as a potential marker for seed germination percentage (Sun et al., 2022b). We also conducted a genome-wide identification of the glutathione reductase (*GR*) genes involved in the AsA-GSH cycle and analyzed its expression pattern during seed germination under stress conditions (Sun et al., 2022a). The objective of this study is to identify the *GLX1*, *GLX2*, and *GLX3* genes in oat at the whole-genome level, and to conduct chromosome mapping, phylogenetic analysis, synteny analysis, conserved domain analysis, *cis*-regulatory element and subcellular localization prediction, and tissue-specific analysis. In addition, the role of these members in seed germination under stress conditions will be analyzed using qPCR. This comprehensive analysis of the glyoxalase gene families in oat will significantly contribute to the genetic enhancement of stress resistance and seed vigor in this essential crop.

## 2 Materials and methods

### 2.1 Identification and chromosomal mapping of AsGLX1, AsGLX2 and AsGLX3 genes

The HMM (Hidden Markov Model) files for the conserved domains of GLX1, GLX2, and GLX3 proteins, including conserved glyoxalase domain (PF00903), metallo-beta-lactamase domain (PF00753), and DJ-1/PfpI domain (PF01965), were obtained from the Pfam database (Mustafiz et al., 2011; Ghosh et al., 2016; Bhowal et al., 2020). The Simple HMM Search module in TBtools software was used to perform the alignment of HMM files to the oat genome (PepsiCo_OT3098_v2_genome, https://wheat.pw.usda.gov/GG3/) to initially identify oat *GLX1*, *GLX2*, and *GLX3* genes. The protein sequences were then submitted to the Pfam website for individual confirmation of their conserved domains, and ultimately determine the members of the GLX1, GLX2, and GLX3 families.

The TBtools software was also used to visualize the chromosome distribution of oat *GLX1*, *GLX2*, and *GLX3* genes (Chen et al., 2020). The nomenclature of oat *GLX1*, *GLX2*, and *GLX3* genes followed the international wheat gene nomenclature rules (http://wheat.pw.usda.gov/ggpages/wgc/98/intro.htm), which could reflect the chromosomal locations of these genes in the oat subgenomes.

### 2.2 Phylogenetic tree, gene duplication, and synteny analysis of AsGLX1, AsGLX2, and AsGLX3 members

The identified AsGLX1, AsGLX2, and AsGLX3 members were aligned with glyoxalase members from *Arabidopsis* and *O. sativa* using ClustalX (v2.1) with default settings (Larkin et al., 2007). The protein sequences of *O. sativa* and *Arabidopsis* GLX1s, GLX2s, and GLX3s used for phylogenetic analysis were sourced from previously published genome-wide identification studies (Mustafiz et al., 2011; Ghosh et al., 2016). The phylogenetic tree was constructed using the neighbor-joining method in MEGA 6.0, with 10,000 bootstrap tests and support values expressed as percentages based on 1000 replications (Tamura et al., 2013). Gene duplication events were analyzed using the Multiple Collinearity Scan toolkit (MCScanX) with default parameters (Wang et al., 2012). To illustrate the interspecies syntenic relationships of *GLX1*, *GLX2*, and *GLX3* genes between oat, *Arabidopsis*, and *O. sativa*, a synteny analysis plot was constructed using Dual Synteny Plotter for MC ScanX in TBtools (Chen et al., 2020).

### 2.3 Physiochemical properties and subcellular localization of AsGLX1s, AsGLX2s, and AsGLX3s

The online program ExPaSy-ProtParam (https://web.expasy.org/protparam/) was utilized to analyze the physical and chemical properties of AsGLX1, AsGLX2, and AsGLX3 proteins, including the amino acid number (AA), molecular weight (MW), and theoretical isoelectric point (pI), instability index (II), negatively charged residues (NCRs), and positively charged residues (PCRs). Protein subcellular localization was predicted using online tools, including WoLF PSORT (https://wolfpsort.hgc.jp/), CELLov.2.5 (http://CELLO.life.nctu.edu.tw/), and Plant-mPLoc (http://www.csbio.sjtu.edu.cn/bioinf/plant-multi/).

### 2.4 Domain architecture of AsGLX1s, AsGLX2s, and AsGLX3s

The TBtools software was used to analyze the protein domains of AsGLX1s, AsGLX2s, and AsGLX3s through HMM files alignment (Chen et al., 2020).

### 2.5 Identification of *cis*-regulatory elements in the promoter *AsGLX1*, *AsGLX2*, and *AsGLX3* genes

To comprehensively investigate the potential response of the *AsGLX1*, *AsGLX2*, and *AsGLX3* genes to stress, we extracted a putative promoter region of 2 kb upstream of the genes from the oat genome sequence and identified *cis*-regulatory elements using the PlantCARE online tool (https://bioinformatics.psb.ugent.be/webtools/plantcare/html/). It may be helpful to further explore the transcriptional regulation mechanism of the glyoxalase genes and reveal its roles in plant development and stress response.

### 2.6 Sampling of different tissues in oat

To perform tissue-specific expression profiling of *AsGLX1*, *AsGLX2*, and *AsGLX3* genes, oat (cv Challenger) samples were collected from seeds, roots, leaves, stems, florets, and lemmas. The seed samples were collected during the imbibition phase (0 h, 12 h, and 24 h) and the development phase (8, 15, and 30 days after flowering, DAF). At the flowering stage, young leaves, lemmas, and florets were collected, while old leaves were obtained from plants at 30 DAF. The roots used in the study were from 10-day-old plants. All seeds used in the study had a germination percentage of 100%.

### 2.7 Stress treatments and sampling during oat seed germination

Seed germination was conducted in a plant growth chamber using plastic petri dishes (11.5 cm × 11.5 cm) containing three layers of filter paper and 50 seeds under a 16-h dark and 8-h light cycle according to the guidelines of the International Seed Testing Association (ISTA, 2019). Normal seed imbibed in distilled water at 20°C was used as the control (CK), while salt, drought, and MG stress treatments employed solutions of 150 mM NaCl (Xu et al., 2021), 20% PEG6000 (Xie et al., 2021), and 10 mM MG (Hoque et al., 2012), respectively. Cold treatment was conducted by imbibing seeds in distilled water at 10°C. Seeds aged for 30 days were also imbibed in distilled water at 20°C. The seed aging treatment was based on the method described by Xia et al. (Xia et al., 2020). Seed samples were collected after 0 h, 6 h, 12 h, 24 h, 36 h, and 72 h of imbibition, with each treatment consisting of three biological replicates, and 20 seeds collected as one replicate.

### 2.8 Gene expression analysis by qRT-PCR and statistical analyses

The total RNA was extracted from oat tissue samples using the Quick RNA isolation Kit (Huayueyang Biotech Co., Ltd., China). The first-strand cDNA was synthesized from 1 μg of RNA using the EasyScript^®^ All-in-One First-Strand cDNA Synthesis SuperMix for qPCR Kit (TransGen Biotech, China). The qRT-PCR was performed on a CFX96 Real-Time System using 2×RealStar Fast SYBR qPCR Mix (Genstar, China), with *AsEIF4A* as the reference gene (Yang et al., 2020). The thermal cycle program was as follows: an initial step at 95°C for 3 min, followed by 40 cycles of 95°C for 15 s and 60°C for 30 s. The relative expression level was determined using the 2^-ΔΔCt^ method.

The comparison of the relative expression levels among different tissues was analyzed using ANOVA and a Duncan’s test in SPSS Statistics 22 and was visualized using GraphPad Prism version 8.0. The expression heatmaps for *AsGLX1*, *AsGLX2*, and *AsGLX3* genes during seed germination under stress were created using TBtools. The significant expression changes were calculated using Student’s *t* test. The details of the primers used in the qRT-PCR assay are listed in **Table S1**.

## 3 Result

### 3.1 Identification and chromosomal mapping of oat glyoxalase genes

**Figure 1:**
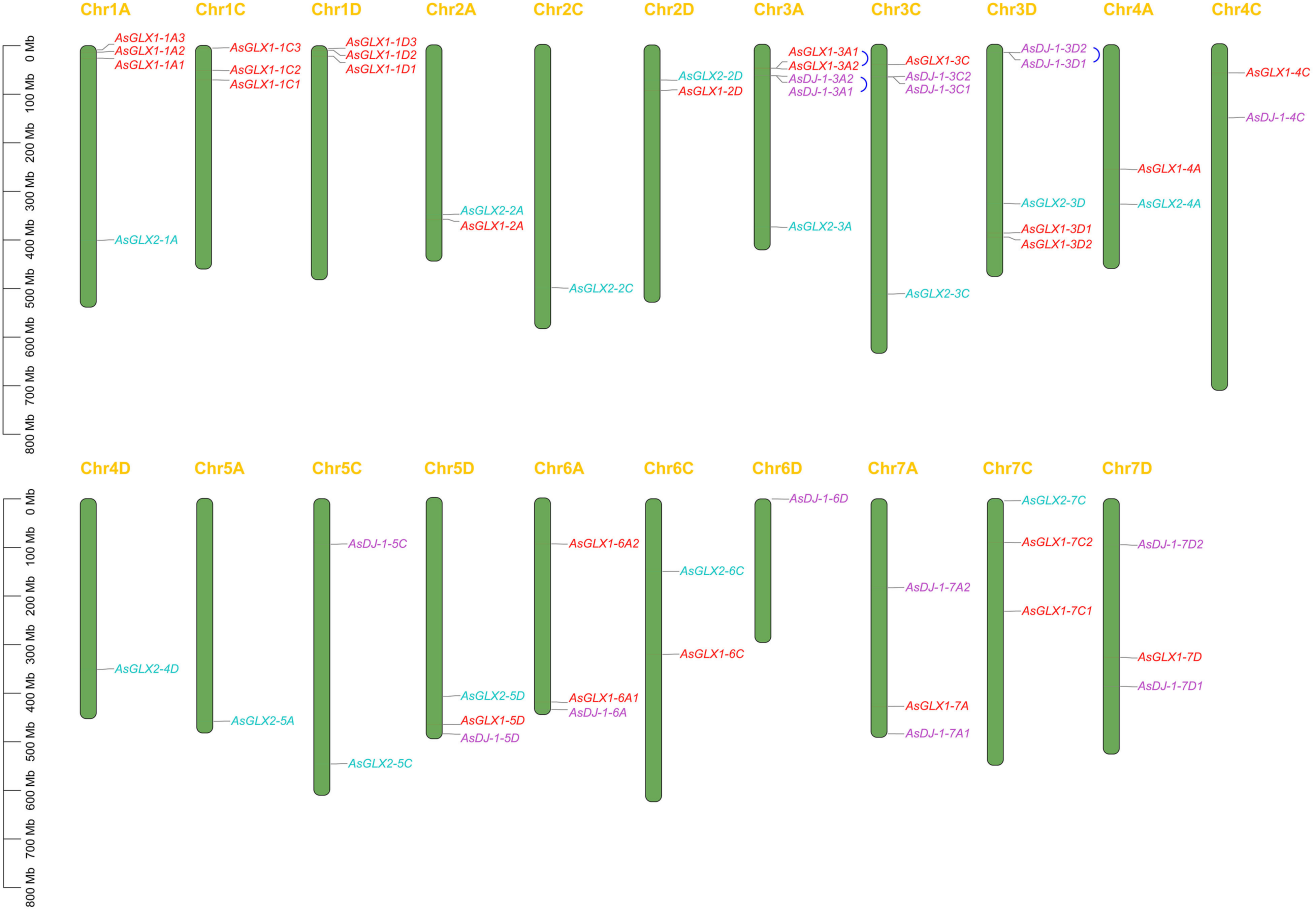
The chromosomal mapping of *AsGLX1*, *AsGLX2*, and *AsGLX3* genes across oat chromosomes. The blue connecting lines represent tandem duplications. The chromosome number is indicated at the top of each chromosome and the scale is shown in megabases (Mb).

Through whole genome alignment in oat, 26 *AsGLX1* genes, 14 *AsGLX2* genes, and 15 *AsGLX3* genes were identified. Chromosomal mapping showed all 26 *AsGLX1* genes were evenly distributed in the A, C, and D subgenomes, with 10 in the A subgenome and 8 in both the C and D subgenomes. *AsGLX1* genes in chromosome set 1 had 9 genes, but only one gene on chromosome set 5. As for 14 *AsGLX2* genes, they were also evenly distributed across the subgenomes, with 5 in both the A and C subgenomes, and 4 in the D subgenome. *AsGLX2* genes were only present once on each chromosome. Regarding *AsGLX3* genes, 5 genes were located in the A subgenome, 4 in the C subgenome, and 6 in the D subgenome. *AsGLX3* genes were not present in chromosome sets 1 and 2, but were highly represented in chromosome sets 3 and 7, with two genes located on each of 3A, 3C, 3D, 7A, and 7D chromosomes. Additionally, some genes of the GLX1 and GLX3 families in oat are adjacent on chromosomes (**Figure 1**).

### 3.2 Phylogenetic analysis of AsGLX1s, AsGLX2s, and AsGLX3s

**Figure 2:**
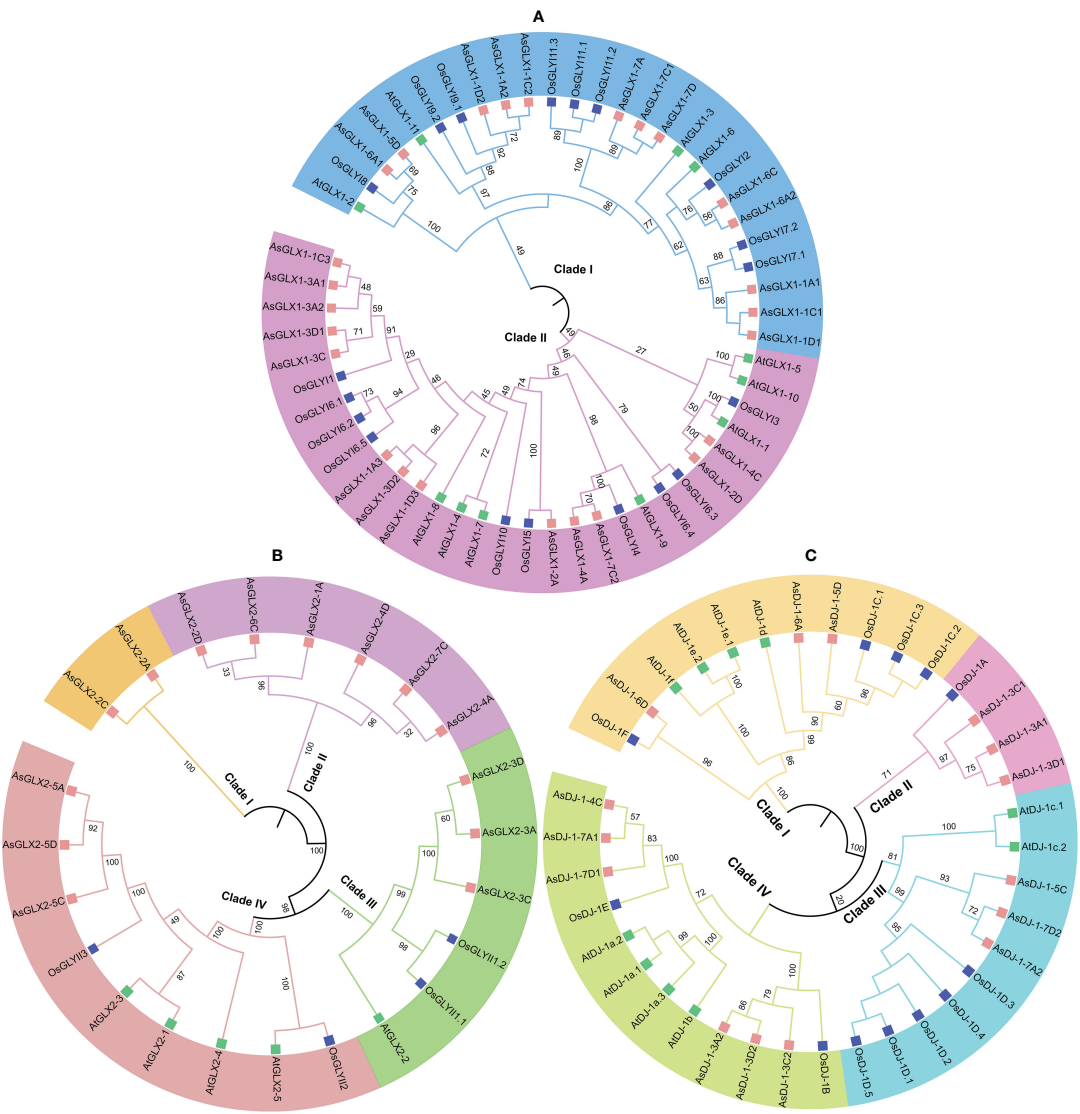
Phylogenetic relationships of GLX1s **(A)**, GLX2s **(B)** and GLX3s **(C)**.

To elucidate the phylogenetic relationships among members of the oat GLX families, amino acid-based phylogenetic trees were constructed using the sequences of GLX1s, GLX2s, and GLX3s from oat, *Arabidopsis*, and *O. sativa* (**Figure 2**). Notably, the number of GLX1 members exceeded that of GLX2 and GLX3 in *A. sativa*, *O. sativa*, and *Arabidopsis*. The 26 AsGLX1s were categorized into two clades. Clade I consisted of 11 AsGLX1s, 4 AtGLX1s, and 5 OsGLX1s, while Clade II contained 16 AsGLX1s, 7 AtGLX1s, and 6 OsGLX1s (**Figure 2A**). The 14 AsGLX2s were classified into four clades, with Clade I and Clade II exclusively containing AsGLX2s, with 2 and 6 members, respectively. Clade III consisted of 1 AtGLX2, 1 OsGLX2s, and 3 AsGLX2s, while Clade IV comprised 3 AsGLX2s, 4 AtGLX2s, and 2 OsGLX2s (**Figure 2B**). Additionally, the 15 AsGLX3 (AsDJ-1) members were also categorized into four clades, with Clade I containing 3 AtDJ-1s, 2 OsDJ-1s, and 3 AsDJ-1s. Clade II included 1 OsDJ-1 and 3 AsDJ-1s, and Clade III consisted of 1 AtDJ-1s, 1 OsDJ-1s, and 3 AsDJ-1s. Clade IV has the largest number of AtDJ-1 members, including 2 AtDJ-1s, 2 OsDJ-1s, and 6 AsDJ-1s (**Figure 2C**).

### 3.3 Gene duplication and synteny analysis of *GLX1*, *GLX2*, and *GLX3* genes

Gene duplication analysis revealed that certain gene pairs of the *GLX1* and *GLX3* gene families, namely *AsGLX1-3A1* and *AsGLX1-3A2*, *AsDJ-1-3A1* and *AsDJ-1-3A2*, and *AsDJ-1-3D1* and *AsDJ-1-3D2*, were the result of tandem duplications, and they were adjacent on the chromosomes. However, no evidence of segmental duplications was found among the genes of the GLX1, GLX2, and GLX3 families (**Figure 1**).

**Figure 3:**
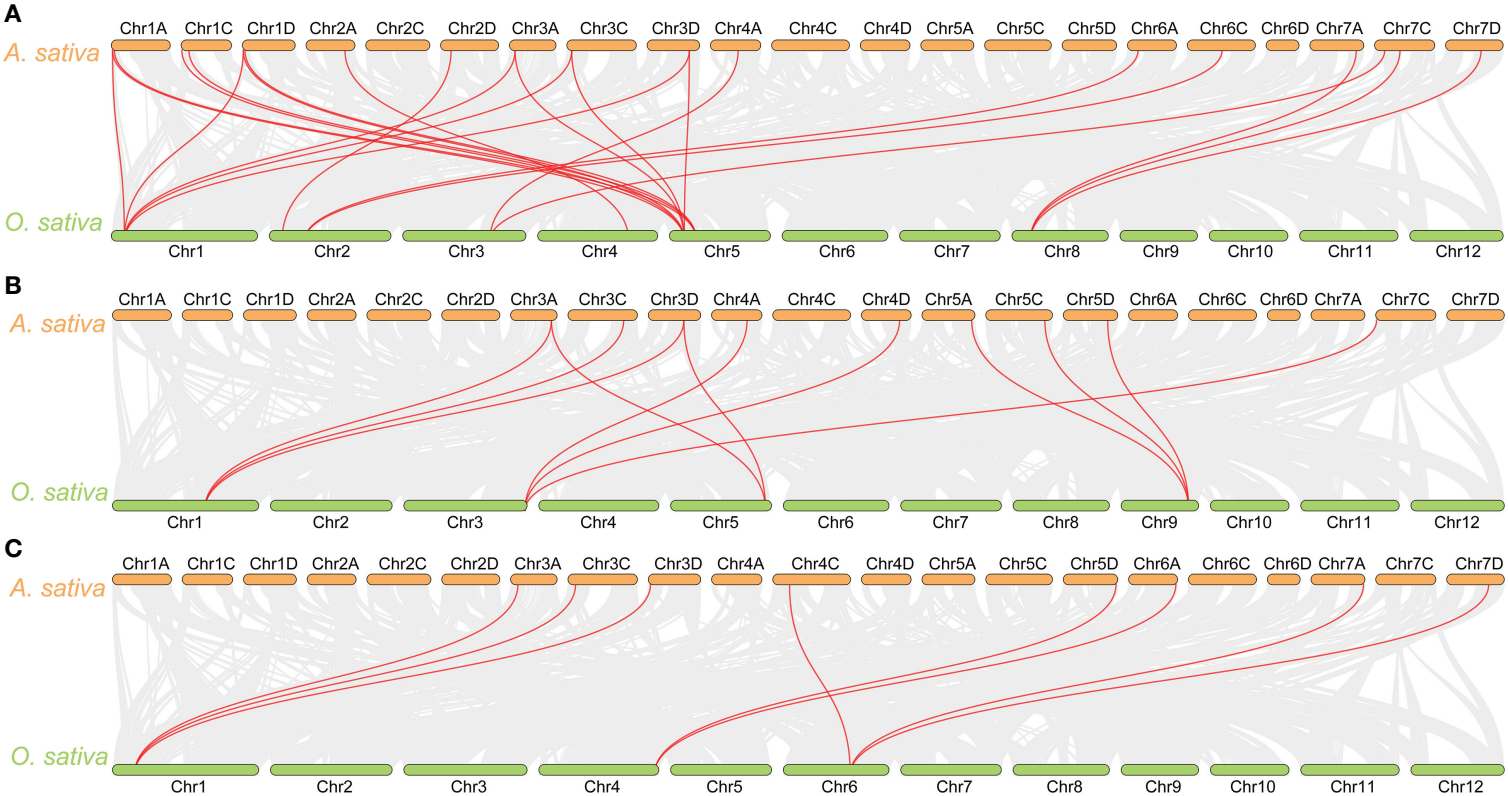
Synteny analysis of *GLX1* **(A)**, *GLX2* **(B)**, and *GLX3* **(C)** genes between *A. sativa* and *O. sativa*. The gray lines show colinear blocks in the genomes of *A. sativa* and *O. sativa*, while the red line highlights the colinear *GLX1*, *GLX2*, and *GLX3* gene pairs.

The synteny analysis between oat and rice, as well as *Arabidopsis*, revealed that there is no collinearity between oat and *Arabidopsis* in the glyoxalase gene families. However, collinearity was observed between oat and rice in the *GLX1*, *GLX2*, and *GLX3* gene families. Specifically, 18 *AsGLX1* genes showed collinearity with 8 *O. sativa* genes, 9 *AsGLX2* genes showed collinearity with 4 *O. sativa* genes, and 8 *AsDJ-1* genes showed collinearity with 3 *O. sativa* genes (**Figures 3A–C**). Among these collinear gene pairs, *OsGLYI6* has the highest number of homologous genes in oat, with six homologous genes (*AsGLX1-1A3*, *AsGLX1-1C3*, *AsGLX1-1D3*, *AsGLX1-3A1*, *AsGLX1-3C*, and *AsGLX1-3D1*). The second highest is *OsGLYI1*, which has five oat homologous genes (*AsGLX1-1A3*, *AsGLX1-1D3*, *AsGLX1-3A1*, *AsGLX1-3C*, and *AsGLX1-3D1*). *OsGLYII3* has three oat homologous genes including *AsGLX2-5A*, *AsGLX2-5C*, and *AsGLX2-5D*. Similarly, *OsDJ-1A* has three homologous genes (*AsDJ-1-3A2*, *AsDJ-1-3C2*, and *AsDJ-1-3D2*), and *OsDJ-1E* also has three oat homologous genes (*AsDJ-1-4C*, *AsDJ-1-7A1*, and *AsDJ-1-7D1*).

### 3.4 Physiochemical properties and subcellular localization of AsGLX1s, AsGLX2s, and AsGLX3s

The online subcellular localization prediction of *AsGLX1*, *AsGLX2*, and *AsGLX3* genes showed that these genes are primarily expressed in the cytoplasm, chloroplasts, and mitochondria (**Table S2**). Based on the predicted results of two or three online servers, 13 genes of the AsGLX1 family were found to be expressed in the cytoplasm, 17 in chloroplasts, and 4 in mitochondria. While, 5 genes of the AsGLX2 family were found to be expressed in the cytoplasm, 8 in chloroplasts, and 7 in mitochondria. Furthermore, 5 genes of the AsGLX3 family were found to be expressed in the cytoplasm, 13 in chloroplasts, and 3 in mitochondria. In addition, *AsGLX1-2A* was predicted by all three online servers to be expressed in the nucleus. Other members, including 6 *AsGLX1s*, 6 *AsGLX2s*, and 4 *AsGLX3s*, predicted by a single program to potentially be expressed in the nucleus, suggest that these members are important for maintaining nuclear stability.

Through the calculation of amino acid length, molecular weight, theoretical isoelectric points, instability index, and charged residues of AsGLX1, AsGLX2, and AsGLX3 proteins, it was found that among the 26 AsGLX1 members, the molecular weight ranged from 15.219 to 94.136 kDa, the amino acid length ranged from 140 AA to 834 AA, and the pI ranged from 4.81 to 8.98. Among them, 12 proteins had an instability index of less than 40, and 20 proteins were rich in negatively charged residues. For the 14 members of AsGLX2, the molecular weight ranged from 32.495 to 143.432 kDa, the amino acid length ranged from 297 AA to 1300 AA, and the pI ranged from 5.87 to 9.12. Among them, 4 proteins had an instability index of less than 40, and 11 proteins were rich in negatively charged residues. As for the 15 members of AsGLX3, the molecular weight ranged from 41.235 to 76.399 kDa, the amino acid length ranged from 395 AA to 723 AA, and the pI ranged from 4.90 to 9.26. Among them, 5 proteins had an instability index of less than 40, and 11 proteins were rich in negatively charged residues (**Table S2**).

### 3.5 Domain analysis of AsGLX1s, AsGLX2s, and AsGLX3s

**Figure 4:**
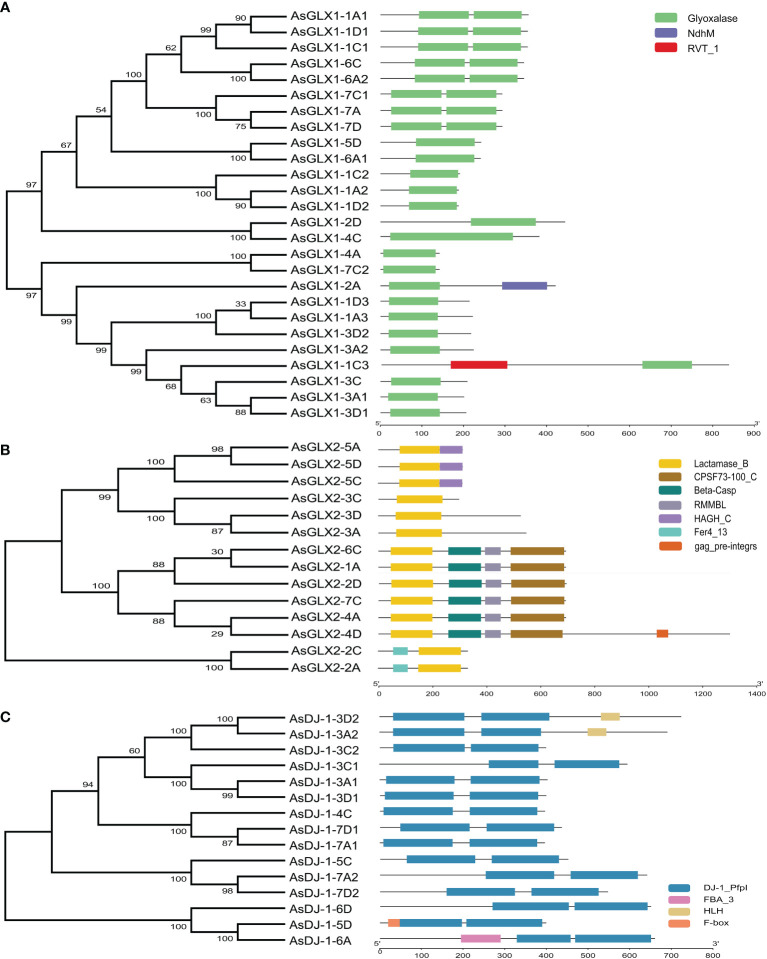
Schematic representation of domain architecture of GLX1s **(A)**, GLX2s **(B)** and GLX3s **(C)** from oat.

Conserved domain analysis of glyoxalase families showed that all AsGLX1s possess conserved glyoxalase domain, AsGLX2s possess conserved metallo-β-lactamase domain, and AsGLX3s possess two conserved DJ-1/PfpI domains (**Figure 4**). Among AsGLX1 members, AsGLX1-1A1, AsGLX1-1C1, AsGLX1-1D1, AsGLX1-6C, AsGLX1-6A2, AsGLX1-7A, AsGLX1-7C1, and AsGLX1-7D possess two conserved glyoxalase domains, with the first domain consisting of 120 aa and the second domain consisting of 115 aa in AsGLX1-1A1, AsGLX1-1C1, AsGLX1-1D1, AsGLX1-6C, and AsGLX1-6A2, while the first domain is 121 aa and the second domain is 120 aa in AsGLX1-7A, AsGLX1-7C1, and AsGLX1-7D. These eight members, along with AtGLYI-3, AtGLYI-6, OsGLYI2, OsGLYI7, and OsGLYI11, belong to Clade I and are Ni^2+^-dependent GLX1s. AsGLX1-5D and AsGLX1-6A1 contain only one glyoxalase domain, with a length of 141 aa, and belong to the same branch in Clade I as AtGLYI-2 and OsGLYI8, indicating that they are Zn^2+^-dependent GLX1s. AsGLX1-1A2, AsGLX1-1D2, and AsGLX1-1C2, which have a domain length of 115 aa, belong to Clade I as well but are not Ni^2+^-dependent GLX1s. The 13 AsGLX1s with functional domains ranging from 108 to 295 aa in Clade II may be GLX1-like proteins. In addition, AsGLX1-1C3 has one reverse transcriptase domain (RVT_1), and AsGLX1-2A has one NADH dehydrogenase complex I subunit M domain (NdhM), which is closely related to its predicted chloroplast localization (**Figures 2A**, **4A**).

AsGLX2-5A, AsGLX2-5C, and AsGLX2-5D belong to Clade IV in the GLX2 family, which are characterized by the presence of both a lactamase B domain (148 aa) and a HAGH_C domain (hydroxyacylglutathione hydrolase C-terminal) with 84 aa. These three members share a conserved metal ion-binding site (THHHYDH). Clade III of the GLX2 family comprises members that contain the lactamase B domain with169 aa, while the lactamase B domain of 6 AsGLX2s in Clade II is 154 aa. AsGLX2s of Clade I, including AsGLX2-2C and AsGLX2-2A, have lactamase B domains that are 156 aa and 158 aa in length, respectively. Addition, the active site of AsGLX2-5A, AsGLX2-5C, AsGLX2-5D, AsGLX2-3A, AsGLX2-3C, AsGLX2-3D, AsGLX2-2C, and AsGLX2-2A all feature the GHT residue, which is essential for catalytic activity. In addition to the lactamase B domain and the HAGH_C domain, some AsGLX2s also contain other domains such as the Pre-mRNA 3’-end-processing endonuclease polyadenylation factor C-term domain (CPSF73-100_C), Beta-Casp domain, Zn-dependent metallo-hydrolase RNA specificity domain (RMMBL), GAG-pre-integrase domain, and 4Fe-4S single cluster domain of Ferredoxin I (Fer4_13) (**Figures 2B**, **4B**).

Although all AsDJ-1s have two DJ-1/PfpI domains, there are differences in the amino acid length that connects the two domains among these members. The number of amino acids between the two domains in Clade I members of the AsDJ-1 family is shorter, ranging from 11 to 14 aa, while the number of amino acids between the two domains in members of the other three Clades is longer, ranging from 39 to 41 aa, except for AsDJ-1-3C2, which has 15 aa. Additionally, AsDJ-1-3A2 and AsDJ-1-3D2 each have one Helix-loop-helix DNA binding domain (HLH), AsDJ-1-6A has one F-box associated domain (FBA_3), and AsDJ-1-5D has one F-box domain (F-box) (**Figures 2C**, **4C**).

### 3.6 C*is*-regulatory elements in the promoter region of glyoxalase genes

**Figure 5:**
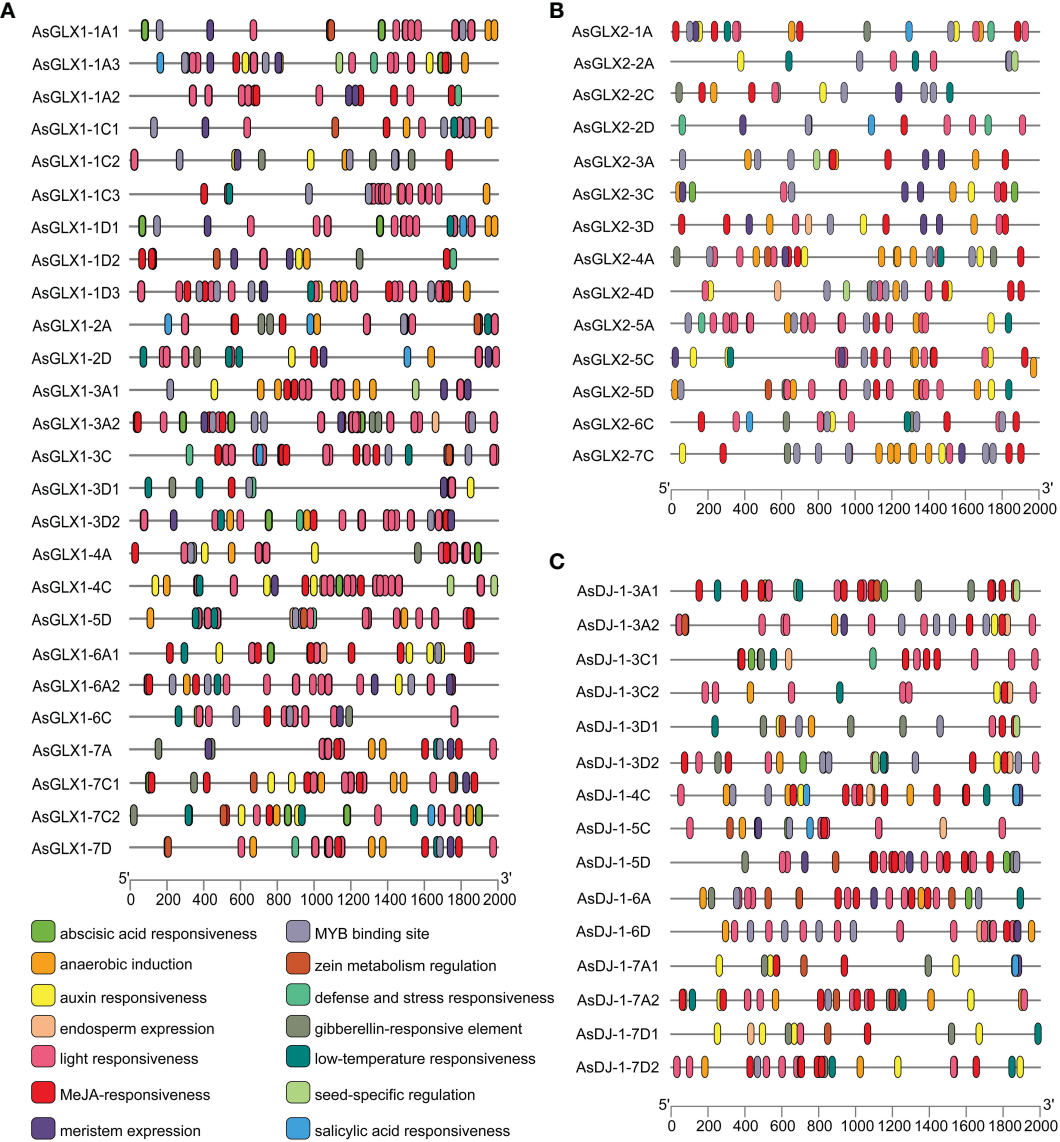
*Cis*-regulatory elements in the promoters of *AsGLX1* **(A)**, *AsGLX2* **(B)**, and *AsGLX3* **(C)** genes.

The analysis of the upstream 2 kb promoter regions of the *AsGLX1*, *AsGLX2*, and *AsGLX3* genes showed that the basic eukaryotic promoter elements CAAT-box and TATA-box are widely distributed in the promoter regions of these genes. Other *cis*-regulatory elements mainly related to hormone responsiveness, anaerobic induction, defense and stress responsiveness, light responsiveness, endosperm expression, MYB binding sites, meristem expression, and seed-specific regulation (**Figure 5**). The hormone responsive elements mainly included ABA, GA, MeJA, SA, and IAA responsiveness. The stress responsiveness was mainly related to low temperature, anaerobic induction, and defense and stress responsiveness. Apart from core elements, hormone responsive elements are the most abundant in the *AsGLX1*, *AsGLX2*, and *AsGLX3* promoters, accounting for 47%, 46%, and 53% of all elements, respectively (**Table S3**). Light responsive elements are also present at high proportions in the *AsGLX1*, *AsGLX2*, and *AsGLX3* gene promoters, accounting for 32%, 24%, and 23%, respectively. Among hormone responsive elements, ABA responsive elements are the most abundant in the *AsGLX1* gene promoters, accounting for about 50%, while MeJA responsive elements are the most abundant in the *AsGLX2* and *AsGLX3* gene promoters, accounting for 46% and 55%, respectively. Elements associated with adverse conditions are present in descending order of number in *AsGLX1*, *AsGLX2*, and *AsGLX3* gene promoters, namely anaerobic induction, low-temperature responsiveness, and defense and stress responsiveness (**Table S3**). Each of the three gene families contains three genes with seed-specific regulatory elements in their promoter regions, namely *AsGLX1-1A3*, *AsGLX1-3A1*, *AsGLX1-4C*, *AsGLX2-2A*, *AsGLX2-3A*, *AsGLX2-4D*, *AsDJ-1-3A1*, *AsDJ-1-3D1*, and *AsDJ-1-3D2*. In addition, MYB binding site elements are also widely distributed in the promoter of genes in the three gene families, particularly in *AsGLX2*, where each gene member’s promoter region contains MYB binding site elements (**Figure 5**).

### 3.7 Tissue-specific expression analysis of *AsGLX1*, *AsGLX2*, and *AsGLX3* genes

**Figure 6:**
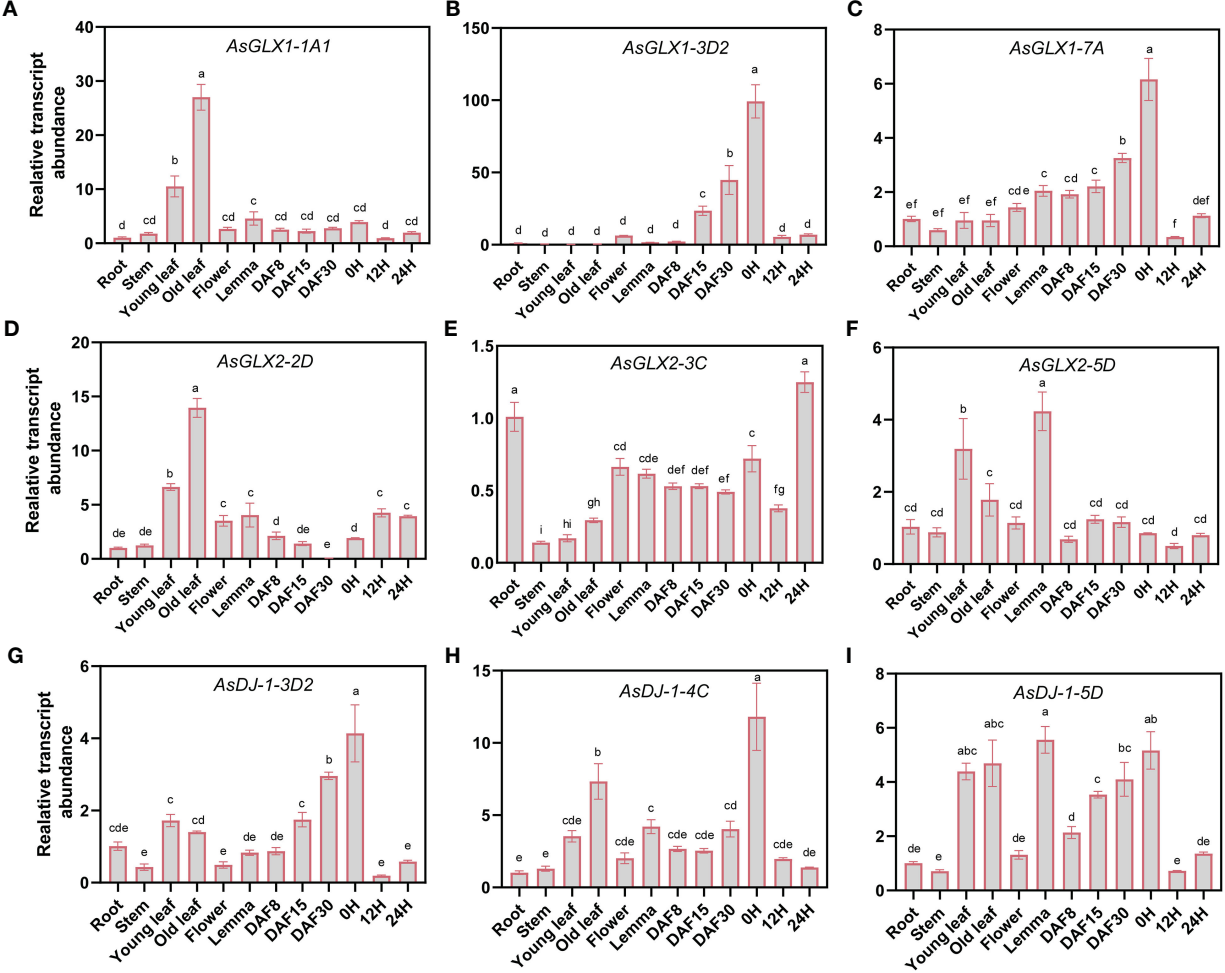
Expression profiling of *AsGLX1, AsGLX2*, and *AsGLX3* genes in different tissues of oat by qPCR. The relative expression was calculated using root as reference. Seeds imbibed for 0 h, 12 h, and 24 h in germination phases were marked as 0 H, 12 H, and 24 H, respectively. Developing seeds at 8, 15, and 30 days after flowering were marked as DAF8, DAF15, and DAF30, respectively. The lower-case letters **(A–I)** represent statistical significance among the samples and the vertical bars represent the ± SEM for three replicates. The mean values sharing different letters, obtained from Duncan test, are different significantly at *p* < 0.05 level.

Three members from each of the *AsGLX1*, *AsGLX2*, and *AsGLX3* gene families were randomly selected in different clades with distinct protein domain compositions and *cis*-element compositions to investigate their tissue-specific expression. *AsGLX1-1A1* and *AsGLX2-2D* showed high expression levels in leaves, particularly in old leaves, and were predicted to be localized in chloroplasts, indicating their importance in leaf function and development (**Figures 6A, D**, **Table S2**). *AsGLX1-7A* and *AsGLX1-3D2* exhibited the highest expression levels in dry seeds, with increasing expression levels during seed development, indicating their potential roles in seed development and dehydration (**Figures 6B, C**). *AsGLX2-3C* was highly expressed in germinated seeds and roots, but had low expression levels in leaves, which is consistent with its predicted mitochondrial localization (**Figure 6E**, **Table S2**). *AsDJ-1-3D2*, *AsDJ-1-4C*, and *AsDJ-1-5D* showed high expression levels in leaves, glumes, dry seeds, and developing seeds, with expression levels gradually increasing during seed development (**Figures 6G–I**). Overall, *AsGLX1*, *AsGLX2*, and *AsGLX3* genes exhibited relatively high expression levels in leaves and seeds, suggesting their potential roles in maintaining leaf function and seed vigor.

### 3.8 Expression analysis of *AsGLX1* genes during seed germination under stresses

**Figure 7:**
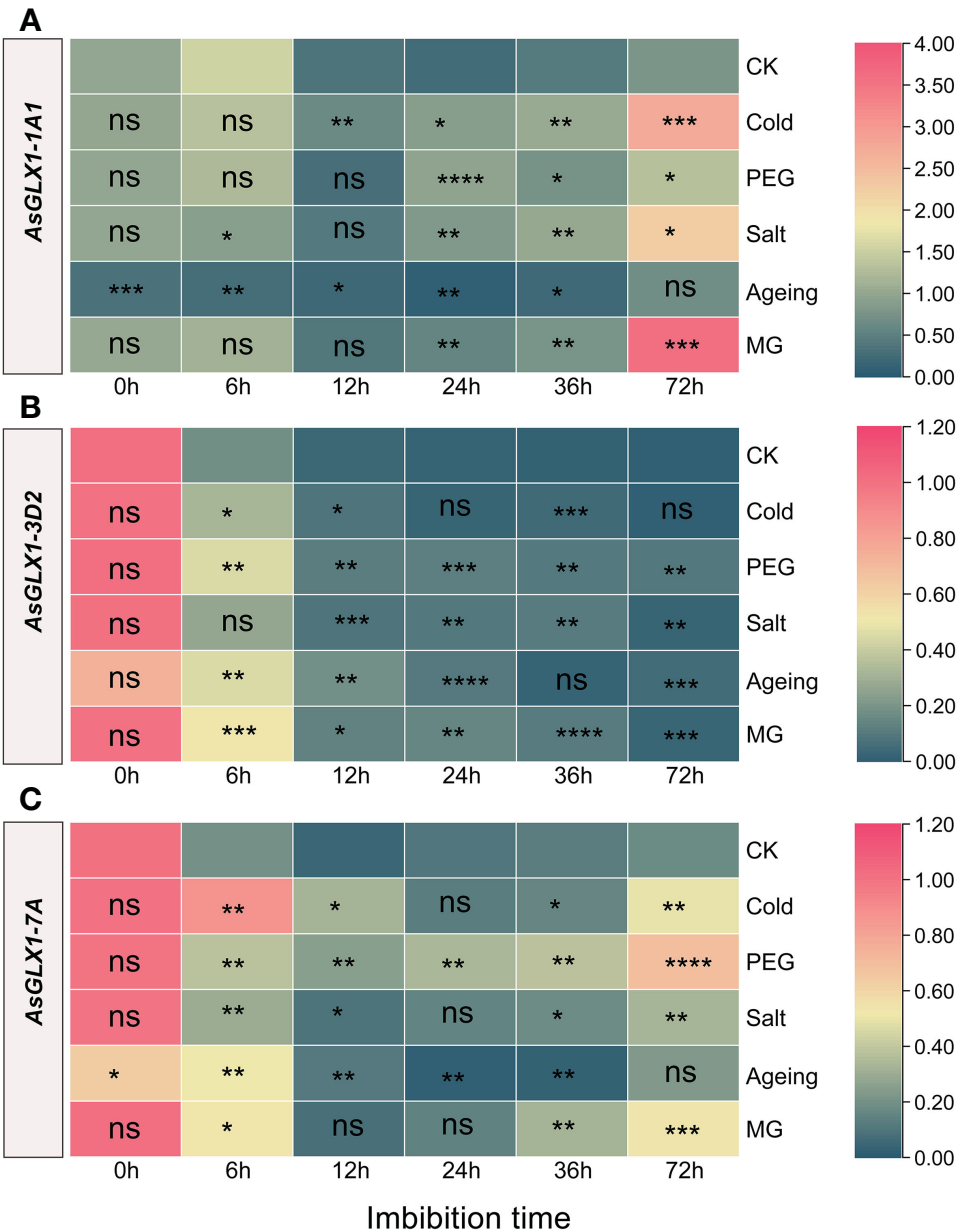
Expression profiling of *AsGLX1-1A1* **(A)**, *AsGLX1-3D2* **(B)** and *AsGLX1-7A* **(C)** during seed germination of oat under stress by qPCR. The relative expression was calculated using dry seed (0 h) of CK as reference. The significant change in comparison to CK has been calculated using Student’s *t* test. *indicates a significant difference at *p* < 0.05; **indicates a significant difference at *p* < 0.01; ***indicates a significant difference at *p* < 0.001; ****indicates a significant difference at *p* < 0.0001; ns represents not significant.

The expression patterns of *AsGLX1-1A1* and *AsGLX1-7A* during seed germination showed a similar trend of first decreasing and then increasing, while *AsGLX1-3D2* exhibited a distinct trend of gradual decrease during germination. After aging treatment, the expression levels of *AsGLX1-1A1* and *AsGLX1-7A* in seeds significantly decreased, while the expression level of *AsGLX1-3D2* showed no significant changes (**Figure 7**, **Supplementary Table 4**).

Compared to the control, *AsGLX1-1A1* showed significant downregulation during 0-36 h imbibition of aged seed, and it showed relatively small changes during the early imbibition stage (0-12 h) under cold, PEG, salt, and MG treatments. However, after 24 h of treatment, *AsGLX1-1A1* expression was significantly induced, particularly under salt, cold, and MG treatments at 72 h of imbibition (**Figure 7A**). *AsGLX1-3D2* exhibited relatively small changes under cold treatment, with no significant difference between the control and treatments at 24 h and 72 h. *AsGLX1-3D2* was upregulated at most imbibition time points during PEG and MG treatments, and in aged seeds, indicating its detoxification role during seed germination under PEG and MG stress and after aging treatment (**Figure 7B**). *AsGLX1-7A* was significantly induced during imbibition for 0-72 h under PEG treatment, with the most significant upregulation occurring at 72 h. However, the expression level of *AsGLX1-7A* in aged dry seeds significantly decreased, being significantly higher than the control during early imbibition (6-12 h), but significantly lower than or not significantly different from the control at 24-72 h of imbibition, suggesting a potential role during the early stages of germination in aged seeds. In addition, *AsGLX1-7A* was significantly induced under cold, salt, and MG treatments at all imbibition time points except for 24 h under cold and salt treatments or 36 h under MG treatment (**Figure 7C**). Overall, *AsGLX1* genes exhibit specificity in response to different stresses, and *AsGLX1-3D2* and *AsGLX1-7A* may play important detoxification roles during seed germination under stress conditions.

### 3.9 Expression analysis of *AsGLX2* genes during seed germination under stresses

**Figure 8:**
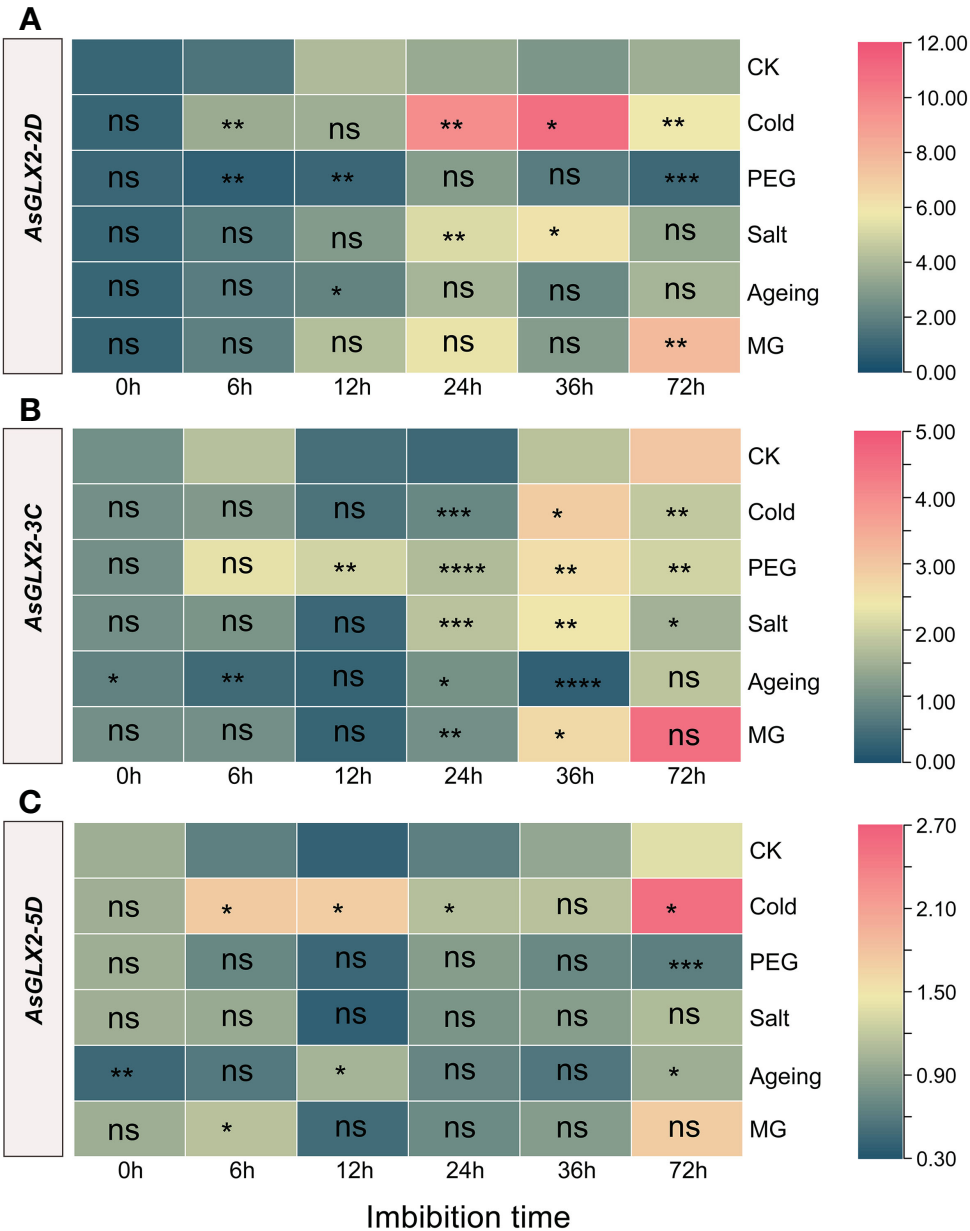
Expression profiling of *AsGLX2-2D* **(A)**, *AsGLX2-3C* **(B)**, *AsGLX2-5D* **(C)** during seed germination of oat under stress by qPCR. The relative expression was calculated using dry seed (0 h) of CK as reference. The significant change in comparison to CK has been calculated using Student’s *t* test. *indicates a significant difference at *p* < 0.05; **indicates a significant difference at *p* < 0.01; *** indicates a significant difference at *p* < 0.001; ****indicates a significant difference at *p* < 0.0001; ns represents not significant.

The response of the three *AsGLX2* genes to stress treatments is relatively smaller compared to the tested *AsGLX1* genes. Following aging treatment, both *AsGLX2-3C* and *AsGLX2-5D* exhibited significant downregulation, while *AsGLX2-2D2* showed no significant difference compared to the CK (**Figure 8**, **Supplementary Table 4**).

*AsGLX2-2D2* was significantly up-regulated during imbibition under cold treatment, except for 12 h, and showed marked response at 24 h and 36 h. In aged seeds, *AsGLX2-2D2* showed almost no significant response. *AsGLX2-2D2* was up-regulated at 24 h and 36 h of imbibition under salt stress, while it was up-regulated at 72 h under MG treatment (**Figure 8A**). *AsGLX2-3C* showed a weak response to different treatments in the early stage of imbibition, and was only significantly up-regulated under PEG treatment for 12 h and significantly down-regulated at 6 h of imbibition in aged seeds. During 24-36 h of imbibition, *AsGLX2-3C* was up-regulated under various treatments, except for significant down-regulation at 36 h of imbibition in aged seeds. At 72 h of imbibition, the expression of *AsGLX2-3C* showed no significant difference compared to the control under various treatments (**Figure 8B**). *AsGLX2-5D* also showed a strong response under cold treatment, and was up-regulated at all-time points except for 36 h of imbibition. Under PEG and salt treatments, *AsGLX2-5D* showed no significant difference in expression level compared to the control at most time points, except for significant down-regulation at 72 h under PEG treatment. *AsGLX2-5D* was significantly up-regulated more than 2-fold at 12 h of imbibition in aged seeds, and its expression level was significantly higher than the control at 6 h of imbibition under MG treatment (**Figure 8C**). Overall, *AsGLX2* genes showed a more significant response to cold treatment, indicating their important role in maintaining seed vigor or promoting seed germination under cold stress.

### 3.10 Expression analysis of *AsDJ-1* genes during seed germination under stresses

**Figure 9:**
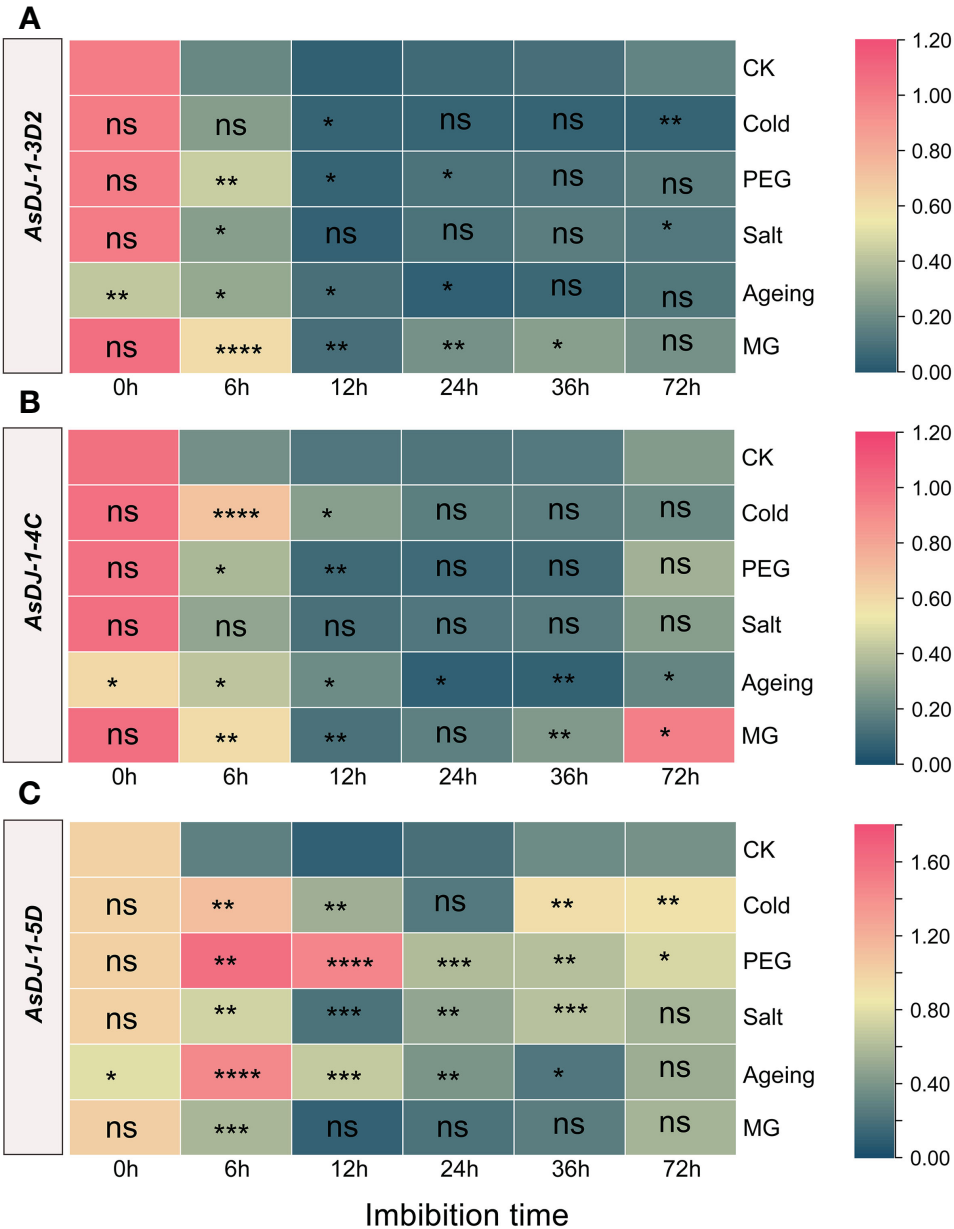
Expression profiling of *AsDJ-1-3D2* **(A)**, *AsDJ-1-4C* **(B)**, and *AsDJ-1-5D* **(C)** during seed germination of oat under stress by qPCR. The relative expression was calculated using dry seed (0 h) of CK as reference. The significant change in comparison to CK has been calculated using Student’s *t* test. *indicates a significant difference at *p* < 0.05; **indicates a significant difference at *p* < 0.01; ***indicates a significant difference at *p* < 0.001; ****indicates a significant difference at *p* < 0.0001; ns represents not significant.

The *AsDJ-1* genes exhibit a diverse response to various stresses during seed imbibition, with *AsDJ-1-5D* displaying a more pronounced response to stress, whereas the responses of *AsDJ-1-4C* and *AsDJ-1-3D2* are comparatively minor. Following aging treatment, all three tested *AsDJ-1* genes show a substantial downregulation (**Figure 9**, **Supplementary Table 4**).

*AsDJ-1-3D2* showed the most significant response under MG treatment, with significant upregulation compared to the control at 6-36 h, and the most significant response occurred at 6 h. Under cold treatment, *AsDJ-1-3D2* was significantly upregulated at 12 h and significantly downregulated at 72 h. It was significantly upregulated at 6-24 h under PEG treatment, and showed a significant response at 12 h and 72 h of imbibition under salt treatment. In aged seeds, it was significantly upregulated during the early imbibition stage (6-12 h) (**Figure 9A**). *AsDJ-1-4C* did not show any significant response under salt treatment. Under cold and PEG treatments, it showed a significant response during the early imbibition stage, and cold treatment significantly induced its expression at 6 h of imbibition. In aged seeds, the expression level of *AsDJ-1-4C* was significantly upregulated during the early imbibition stage (6-12 h), and then showed a significant decrease. Moreover, under MG treatment, its expression level was significantly upregulated at 6 h, 36 h, and 72 h of imbibition (**Figure 9B**). *AsDJ-1-5D* was significantly upregulated during the imbibition under PEG treatment, especially in the early imbibition stage (6-12 h). There was no significant response under cold treatment at 24 h and under salt treatment at 72 h. At other time points, both cold and salt treatments significantly induced the expression of *AsDJ-1-5D*. During the imbibition of aged seeds, *AsDJ-1-5D* was significantly upregulated during 6-24 h, with the most significant response observed at 6 h, with an increase of more than 5 folds. Under MG treatment, its expression level was only significantly upregulated at 6 h (**Figure 9C**). Overall, *AsDJ-1-5D* has a relatively strong role in detoxification during the early germination stage of aged seeds and under PEG treatment.

## 4 Discussion

The glyoxalases participate in various biological processes in plants (Singla-Pareek et al., 2020), such as stress response (Kaur et al., 2014), seed germination (Schmitz et al., 2017), plant senescence (Singla-Pareek et al., 2009), nutrient regulation (Borysiuk et al., 2022), signal transduction (Sankaranarayanan et al., 2017), starch synthesis (You et al., 2019), and pollen development (Sankaranarayanan et al., 2015). Their primary role in plants is to detoxify MG, which is spontaneously produced in plants and significantly accumulates under abiotic stresses such as salinity, drought, heavy metals, and low temperature, thus hindering plant growth and development (Kaur et al., 2014; Hasanuzzaman et al., 2017; Sankaranarayanan et al., 2017). Higher plants often contain multiple members of the *GLX1*, *GLX2*, and *GLX3* gene families, and different members exhibit variations in their subcellular localization, expression patterns, and functional roles (Ghosh and Islam, 2016; Schmitz et al., 2017; Singla-Pareek et al., 2020). Therefore, a comprehensive genome-wide identification of glyoxalase gene families and understanding their chromosome distribution, evolutionary relationships, conserved domains, *cis*-regulatory elements, and gene expression patterns is crucial for exploring the functional diversity of glyoxalase genes and improving plant stress resistance.

The GSH-dependent pathway is the main pathway for clearing MG by GLX1 and GLX2 enzymes, which has led previous studies on plant glyoxalases to focus mainly on these two enzymes. However, the GSH-independent GLX3 has only recently received attention. Genome-wide identification and analysis of GLX1 and GLX2 families have been completed in in various plants. In *Arabidopsis*, a total of 11 *GLX1* genes and 5 *GLX2* genes were identified, while 11 *GLX1* genes and 3 *GLX2* genes were found in *O. sativa* (Mustafiz et al., 2011). In *G. max*, 24 *GLX1* genes and 12 *GLX2* genes were identified (Ghosh and Islam, 2016). In *S. bicolor*, 15 *GLX1* genes and 6 *GLX2* genes were found (Bhowal et al., 2020). However, the extensive whole-genome identification of glyoxalase families has ignored *GLX3* genes, and only a few plants, such as *Medicago truncatula* and *V. vinifera*, have been systematically identified for the GLX1, GLX2, and GLX3 families (Ghosh, 2017; Li et al., 2019). In addition, recent studies have also individually identified GLX3s in some plant species. For example, 217 GLX3s were obtained by using AtDJ-1d (AT3g02720) to compare the Swiss-Prot database, including 8 *Oryza* species, 2 *Triticum* species, *Hordeum vulgare*, *Zea mays*, *S. bicolor*, *Setaria italica*, and *Brachypodium distachyon*. Among them, 12 GLX3s were identified in *O. sativa*, which were encoded by 6 genes and strongly induced by MG (Ghosh et al., 2016). To gain insight into the evolutionary patterns of GLX3, the evolution of GLX3s across prokaryotes and eukaryotes were studied, and 183 GLX3s belonging to 69 species was used for evolutionary analysis in plants (Kumar et al., 2021). In summary, the study of plant GLX3 have become a focus and hotspot, and systematic analysis of GLX1, GLX2, and GLX3 members will play an important role in plant improvement.

A total of 26 *AsGLX1* genes, 14 *AsGLX2* genes, and 15 *AsGLX3* genes were identified in oat. The number of *AsGLX1* and *AsGLX2* genes identified in *Arabidopsis*, *O. sativa*, *G. max*, *S. bicolor*, and *B. rapa* was lower than that in oat, which may be related to genome duplication events during evolution (Ghosh and Islam, 2016; Yan et al., 2018; Bhowal et al., 2020; Peng et al., 2022). In the secondary branches of the evolutionary tree of the GLX1, GLX2, and GLX3, there were two or three oat homologous genes, originating from two or three oat subgenomes (A, C, and D), corresponding to one *Arabidopsis* glyoxalase gene. Oat has diploid and tetraploid ancestors, and genome duplication may be the main reason for the high number of homologous genes in oat (Peng et al., 2022). Some clades contained only GLX members from *O. sativa* and oat, such as OsGLYI11, AsGLX1-7A, AsGLX1-C1, and AsGLX1-D in GLX1, as well as OsDJ-1A, AsDJ-1-3A1, AsDJ-1-3C1, and AsDJ-1-3D1 in GLX3, indicating that the three families evolved asynchronously in the *O. sativa*, oat, and *Arabidopsis* genomes. Furthermore, the gene duplication events analysis of *AsGLX1*, *AsGLX2*, and *AsGLX3* genes showed that only *AsGLX1* and *AsGLX3* gene families had tandem duplications, while none of the three families had segmental duplications. In contrast, in *O. sativa*, *Arabidopsis*, *B. napus* and *G. max*, gene duplications of *GLX1* and *GLX2* genes were caused by the segmental duplication, rather than tandem duplication (Mustafiz et al., 2011; Ghosh and Islam, 2016; Yan et al., 2023). Therefore, there are significant differences in gene duplication events that occur in the glyoxalase families in different plant species.

The AsGLX1 family members, AsGLX1-1A1, AsGLX1-1C1, AsGLX1-1D1, AsGLX1-6C, AsGLX1-6A2, AsGLX1-7A, AsGLX1-7C1, and AsGLX1-7D, contain two conserved glyoxalase domains and are clustered with the Ni^2+^-dependent GLX1 members in *Arabidopsis*, indicating that they are Ni^2+^-dependent AsGLX1s. AsGLX1-5D and AsGLX1-6A1 contain a single glyoxalase domain of 141 amino acids in length and are clustered with Zn^2+^-dependent GLX1s in *Arabidopsis* and *O. sativa*, indicating that they are Zn^2+^-dependent AsGLX1s (Ghosh and Islam, 2016; Bhowal et al., 2020). The activity of GLX1 depends on divalent metal ions, and early studies suggested that the type of divalent ion required for GLX1 activity varies between prokaryotes and eukaryotes. GLXI in humans and yeast is Zn^2+^-dependent, while GLX1 in *E. coli* requires Ni^2+^ for optimal activity (He et al., 2000). However, studies on plants have identified two types of divalent ion-dependent GLX1s. For instance, in *Arabidopsis*, AtGLYI2 is dependent on Zn^2+^, whereas AtGLYI3 and AtGLYI6 are dependent on Ni^2+^. Most studies on the function of plant GLX1s have focused on these two types, with less research on other GLX1-like proteins. In *O. sativa*, OsGLYII-1 has ethylmalonic encephalopathy-1 activity, which can be activated by Ca^2+^, and OsGLYII-2 has a binuclear zinc/iron center at its active site that is crucial for its activity. AsGLX2-5A, AsGLX2-5C, and AsGLX2-5D have both lactamase B and HAGH_C domains, all of which contain conserved THHHYDH metal ion-binding sites and GHT activity sites, indicating that these members encode for putative functionally active AsGLX2 enzymes (Ghosh and Islam, 2016; Bhowal et al., 2020; Singla-Pareek et al., 2020). GLX2 enzymes with these two domains, including the THHHYDH and GHT sites, have been identified in other plants such as sorghum (SbGLYII-3 and SbGLYII-4) and grape (VvGLYII-like1 and VvGLYII-like2)(Ghosh and Islam, 2016; Li et al., 2019).

Subcellular localization prediction analysis revealed that AsGLX1s are mainly expressed in the cytoplasm, chloroplasts, and mitochondria, AsGLX2s are mainly expressed in chloroplasts and mitochondria, and AsGLX3s are mainly expressed in chloroplasts. Chloroplasts and mitochondria are organelles responsible for photosynthesis and respiration, respectively, and are metabolically active. They are not only the main source of ROS, but also the potential organelles for MG production (Hasanuzzaman et al., 2017). Excessive MG has been found to inhibit photosynthesis and disrupt mitochondrial function (Kaur et al., 2016). Under stress conditions, chloroplast and mitochondrial components are often hotspots for glycation (Tripathi et al., 2023). The expression and activity of glyoxalase enzymes in chloroplasts and mitochondria are crucial for protecting the photosynthetic system and mitochondrial function. In addition, there are also some members that function in the nucleus, which may be important for protecting the nucleus and maintaining DNA and RNA stability. For example, in sorghum, SbGLYI-8/8.1 proteins were also found to harbor putative nuclear localization signals and therefore, may catalyze the conversion of nuclear MG to SLG (Bhowal et al., 2020). And OsGLYI-8 is located in the nucleus and can alleviate DNA damage caused by MG in the nucleus (Kaur et al., 2017).

Through tissue-specific expression analysis of some members, it was found that their expression patterns were consistent with subcellular localization predictions. For example, *AsGLX1-7A* was predicted to be expressed in the cytoplasm and was found to be highly expressed in developing seeds and dry seeds. *AsGLX2-3C* was predicted to be primarily expressed in mitochondria and was found to be highly expressed in roots, flowers, dry seeds, and seeds imbibed for 24 h. *AsDJ-1-4C* was predicted to be expressed in chloroplasts and cytoplasm and was found to be highly expressed in dry seeds and leaves. Overall, most of the tested members were expressed in leaves and dry seeds, indicating their important roles in maintaining leaf function and seed vigor. *AsGLX1-3D2* is exclusively expressed in developing and dry seeds, indicating that its function may be seed-specific, such as enhancing seed tolerance to stress during seed maturation and dehydration. In *Arabidopsis* and *O. sativa*, *AtGLYI8*, *OsGLYI3*, and *OsGLYI10* were also found to be highly expressed in developing seeds (Mustafiz et al., 2011). Glyoxalases have been suggested play an important role in seed development, seed germination, and seed vigor regulation. In *Arabidopsis*, a cytosolic GLXI3 isoform works on the elimination of toxic reactive carbonyl species during germination and seedling establishment (Schmitz et al., 2017). And the lack of *AtGLYI2* resulted in severe inhibition of seed germination under MG treatment, and the growth of seedlings was also limited under salt stress (Liu et al., 2022). In rice, OsGLYI7 participates in starch synthesis in the endosperm, and its mutant had significantly reduced starch content and altered expression of starch synthesis genes (You et al., 2019). While, *OsGLYI3* is specifically expressed in rice seeds and contributes to seed longevity and salt stress tolerance (Liu et al., 2022). *AsGLX2-3C* is highly expressed in roots and flowers, showing a significant difference from other members, and may play a detoxifying role during root and flower development. In *B. napus*, GLX1 is required for pollination and is targeted by the self-incompatibility system (Sankaranarayanan et al., 2015). Previous studies have mainly neglected the role of the glyoxalases in seeds. The diverse expression patterns and subcellular localization of *GLX1*, *GLX2*, and *GLX3* genes provide a basis for their functional diversity in plants, and their important roles in seeds should be given more attention in the future.

The expression patterns of glyoxalase gene families, especially *GLX1* and *GLX2* genes, under stresses have been extensively studied. It has proven GLX1 and GLX2 transcript levels and enzyme activities could be induced by various adverse conditions (Kaur et al., 2014; Sankaranarayanan et al., 2017). But their expression patterns during seed germination have been rarely reported. However, from seed sowing to seedling establishment stage, crops often suffer from stress such as drought, low temperature, or salinity, which can lead to a significant reduction in crop yield. Therefore, analyzing the expression patterns of *GLX1*, *GLX2*, and *GLX3* genes during seed germination under stress conditions is crucial for understanding their role in regulating seed vigor and coping with stress. In this study, it was found that the *GLX1* genes *AsGLX1-3D2* and *AsGLX1-7A* may play an important role in detoxification during seed germination under stress conditions. They were significantly induced by cold, drought, salt, MG, and aging treatments in the early stages of germination (6 h and 12 h). *AsGLX2-5D* was upregulated in the early stages of germination by cold, MG, and aging induction, while *AsGLX2-3C* was induced by various stresses in the later stages (24h-72h) of imbibition, indicating that these genes may play a detoxification role during seed germination under stress conditions, but with specificity in terms of stress time and types. GLX1 and GLX2 transgenic plants often exhibit increased stress resistance, while their function-deficient mutants exhibit reduced stress resistance (Kaur et al., 2014; Kaur et al., 2016; Sankaranarayanan et al., 2017). For instance, in *Arabidopsis*, complementation of *AtGLYI2* and the rice homolog *OsGLYI8* significantly enhanced stress resistance in the *atglyI-2* mutant (Kaur et al., 2017). Overexpression of *OsGLYII3* in tobacco significantly improved plant resistance to MG and NaCl (Singla-Pareek et al., 2003). Overexpression of *OsGLYII2* in *E. coli* and tobacco resulted in a significant increase in resistance to both MG and salt stresses, along with improvements in plant photosynthetic performance and a decrease in oxidative damage (Ghosh et al., 2014). The expression analyzed GLX3 members, *AsDJ-1-3D2*, *AsDJ-1-4C*, and *AsDJ-1-5 AsDJ-1-5D*, show significant responses to stress during the early stages of seed imbibition, with *AsDJ-1-5 AsDJ-1-5D* being significantly upregulated throughout the imbibition process under stress conditions such as cold, drought, salt, and aging. In both *E. arundinaceus* and commercial sugarcane hybrids, *Gly III* respond to drought and salt stress like *Gly II* and *Gly I*, but the expression level of *Gly III* is higher under stress (Manoj et al., 2019). Additionally, the overexpression of *EaGly III* in sorghum confers significant improvements in drought and salt stress resistance, as evidenced by increased levels of proline and soluble sugars, enhanced photosynthetic and antioxidant abilities, and decreased lipid peroxidation in transgenic lines (Mohanan et al., 2020; Mohanan et al., 2021). Although studies on *GLX3* genes in plants are limited, they all indicate the great potential of *GLX3* in combating unfavorable conditions.

In this study, it was found that AsGLX1-7A has two glyoxalase domains and is a Ni^2+^-dependent GLX1, located in the cytoplasm, and has a high gene expression level in seeds. AsGLX2-5D has THHHYDH metal ion and GHT active sites and may play a protective role in mitochondria, which are important for respiration and stress responses during seed germination. AsDJ-1-5D has two conserved DJ-1 domains and a high gene expression level in seeds. The three genes showed significant responses to various stresses and encode for putative functionally active glyoxalase enzymes. In addition, *AsGLX1-3D2* is seed-specifically expressed, and *AsGLX1-2A* may function in the protection of nucleic acid stability under stress conditions. These members of the glyoxalase gene families can be focused on in future research as potential candidate genes for oat stress resistance breeding and seed vigor improvement.

## 5 Conclusion

The identification of oat genes involved in stress resistance and seed vigor regulation represents a crucial endeavor in oat molecular breeding, with significant implications for crop yield and germplasm conservation. This study found that oat has more glyoxalase genes than most other plant species due to genome duplication events and tandem duplications during evolution. These genes are generally regulated by hormones and respond to adverse conditions. Their diverse tissue expression patterns and subcellular localizations indicate their functional diversity in plants, especially in leaf development and seed vigor formation. *AsGLX1-3D2* was specifically expressed in seeds, and *AsGLX1-2A* may play an important role in alleviating nucleic acid glycation. In addition, AsGLX1-7A has potential Ni^2+^-dependent GLX1 activity, and AsDJ-1-5D has double DJ-1 domains, both of which can significantly respond to cold, drought, salt, MG and aging treatments. AsGLX2-5D has potential GLX2 activity and can respond to cold, aging, and MG stress. These highlighted genes are promising candidates for further investigation into oat stress resistance or seed vigor regulation. However, the specific functions of other members of AsGLX1, AsGLX2, and AsGLX3 families require further investigation. Nonetheless, such research may lead to the development of new strategies for breeding oats with improved stress tolerance and seed vigor through the utilization of glyoxalase genes.

## Data availability statement

The datasets presented in this study can be found in online repositories. The names of the repository/repositories and accession number(s) can be found in the article/**Supplementary Material**.

## Author contributions

PM conceived and designed the experiment. MS and SS performed the experiments and analyzed the data. ZJ, CO, JW, HZ, and WM contributed to the experiment. MS and SS wrote the paper, and ML and PM revised the paper. All authors contributed to the article and approved the submitted version.
