# Supplementary material for: Genome-wide analysis and expression profiling of glyoxalase gene families in oat (Avena sativa) indicate their responses to abiotic stress during seed germination
Source: Front Plant Sci. 2023 Jun 15;14:1215084. doi: 10.3389/fpls.2023.1215084 (PMC10308377; doi:10.3389/fpls.2023.1215084)
Supplement: Supplementary file 1 [file Table_1.docx]

**Supplementary Table 1.** Primer information of *AsGLX1, AsGLX2 and AsGLX3* genes for qRT-PCR analysis.

| No. | Gene name | Primer sequence (5′-3′) | Product size (bp) | Tm (°C) |
| --- | --- | --- | --- | --- |
| 1 | *AsGLX1-1A1* | TGGCGTGGAAAGTTATGACATTGG | 125 | 59.7 |
|  |  | CTGGACCTGGTTCTCTTGTTACTG |  | 58.7 |
| 2 | *AsGLX1-3D2* | AAGCGGTGCGTGGAGGAG | 80 | 58.8 |
|  |  | ATGGTGGCGAATGTCTGGTC |  | 57.3 |
| 3 | *AsGLX1-7A* | ATGACGACGAAGATAAGACCACTG | 132 | 58.3 |
|  |  | CGACAGCCTCAGCACTCTTG |  | 58.0 |
| 4 | *AsGLX2-2D* | TGGCATACGCTTCTGGTGTTATAC | 117 | 58.9 |
|  |  | CGGTCTTCTTCACGGGAGTAGTC |  | 60.0 |
| 5 | *AsGLX2-3C* | AGGCTCCTCTTCAGGCAACTATTC | 92 | 60.0 |
|  |  | AACACGGCGGGCTTCTCG |  | 59.5 |
| 6 | *AsGLX2-5D* | GGTGGCAATTTGGAACTGAAGG | 108 | 57.7 |
|  |  | CGTGTGACCATCGCTTAGGG |  | 57.8 |
| 7 | *AsDJ-1-3D2* | AGGGTGGTGGTGGATAGAAATGC | 139 | 60.1 |
|  |  | GTTGAGGGCGAACATACAAAGGTC |  | 60.0 |
| 8 | *AsDJ-1-5D* | GGAGCCAGCGAGCAAGCG | 127 | 60.6 |
|  |  | GCAGACGGAGCGGAAGCG |  | 60.3 |
| 9 | *AsDJ-1-4C* | TGCCCAAGCGTATGCCAAATC | 144 | 59.1 |
|  |  | GATGTAGCCTTCTTTCCCTTCAGC |  | 59.3 |
