# Supplementary material for: Genome-wide analysis and expression profiling of glyoxalase gene families in oat (Avena sativa) indicate their responses to abiotic stress during seed germination
Source: Front Plant Sci. 2023 Jun 15;14:1215084. doi: 10.3389/fpls.2023.1215084 (PMC10308377; doi:10.3389/fpls.2023.1215084)
Supplement: Supplementary file 2 [file Table_2.docx]

**Supplementary Table 2.** Physiochemical properties and subcellular localization of AsGLX1s, AsGLX2s, and AsGLX3s.

| **Gene Names** | **Gene ID** | **AA** | **MW** | **pI** | **II** | **NCR/PCR** | **CELLO** | **WoLF PSORT** | **Plant-mPLoc** |
| --- | --- | --- | --- | --- | --- | --- | --- | --- | --- |
| *AsGLX1-1A1* | AVESA.00001b.r1.1Ag0000186.1 | 354 | 38.873 | 5.78 | 34.55 | 49/45 | Ch | Ch | Cyt |
| *AsGLX1-1A2* | AVESA.00001b.r1.1Ag0000107.1 | 291 | 32.491 | 5.23 | 23.66 | 45/37 | Cyt | Cyt, Cysk, Ch, Nu | Cyt |
| *AsGLX1-1A3* | AVESA.00001b.r1.1Ag0000067.1 | 220 | 23.897 | 5.44 | 68.31 | 23/16 | Cyt | Ch | Ch, Cyt |
| *AsGLX1-1C1* | AVESA.00001b.r1.1Cg0000632.1 | 352 | 38.666 | 5.63 | 34.06 | 49/44 | Mt, Ch, Cyt | Mt, Ch | Cyt |
| *AsGLX1-1C2* | AVESA.00001b.r1.1Cg0000538.1 | 189 | 20.885 | 8.75 | 39.42 | 20/23 | Mt | Mt, Ch | Cyt |
| *AsGLX1-1C3* | AVESA.00001b.r1.1Cg0000038.1 | 834 | 94.136 | 7.27 | 47.91 | 92/92 | PM | Cyt | Ch |
| *AsGLX1-1D1* | AVESA.00001b.r1.1Dg0000169.1 | 352 | 38.718 | 6.19 | 33.33 | 48/46 | Mt, Ch | Ch | Cyt |
| *AsGLX1-1D2* | AVESA.00001b.r1.1Dg0000090.1 | 186 | 20.56 | 8.81 | 36.93 | 20/23 | Mt | Mt, Ch | Ch, Cyt |
| *AsGLX1-1D3* | AVESA.00001b.r1.1Dg0000047.1 | 212 | 23.144 | 5.61 | 68.94 | 23/17 | Cyt | Ch, Cyt, PM, Nu | Ch, Cyt |
| *AsGLX1-2A* | AVESA.00001b.r1.2Ag0001617.1 | 420 | 47.235 | 6.1 | 60.71 | 52/47 | Nu | Ch, Cyt, Mt, Nu | Ch, Cyt, Nu |
| *AsGLX1-2D* | AVESA.00001b.r1.2Dg0000984.1 | 442 | 47.128 | 5.46 | 46.36 | 50/39 | Ch | Cyt | Cyt |
| *AsGLX1-3A1* | AVESA.00001b.r1.3Ag0000549.1 | 199 | 21.352 | 5.28 | 61.03 | 22/15 | Ch | Ch, Cyt | Ch |
| *AsGLX1-3A2* | AVESA.00001b.r1.3Ag0000550.1 | 222 | 24.013 | 5.35 | 67.95 | 25/19 | Ch | Ch, Cyt | Ch |
| *AsGLX1-3C* | AVESA.00001b.r1.3Cg0000456.1 | 207 | 22.219 | 5.64 | 65.12 | 22/17 | Ch, Cyt | Ch, Cyt | Ch |
| *AsGLX1-3D1* | AVESA.00001b.r1.3Dg0002056.1 | 204 | 21.832 | 5.59 | 61.8 | 22/16 | Ch | Ch, Cyt | Ch |
| *AsGLX1-3D2* | AVESA.00001b.r1.3Dg0002150.1 | 216 | 23.545 | 5.32 | 69.78 | 23/16 | Ch, Cyt | Ch | Cyt |
| *AsGLX1-4A* | AVESA.00001b.r1.4Ag0001083.1 | 140 | 15.233 | 5.47 | 55.53 | 17/13 | Cyt | Ch, Cyt, Mt, Nu | Cyt |
| *AsGLX1-4C* | AVESA.00001b.r1.4Cg0000786.1 | 380 | 40.678 | 4.81 | 39.8 | 54/31 | Cyt | Ch, Cyt, Mt, Po | Cyt |
| *AsGLX1-5D* | AVESA.00001b.r1.5Dg0003058.1 | 240 | 26.752 | 8.98 | 44.88 | 28/32 | Mt, Ch | Mt, Ch | Cyt |
| *AsGLX1-6A1* | AVESA.00001b.r1.6Ag0002884.1 | 239 | 26.532 | 8.93 | 50.61 | 28/32 | Mt, Ch | Ch | Cyt |
| *AsGLX1-6A2* | AVESA.00001b.r1.6Ag0000540.1 | 343 | 37.358 | 5.71 | 33.54 | 47/44 | Ch | Ch | Cyt |
| *AsGLX1-6C* | AVESA.00001b.r1.6Cg0002317.1 | 343 | 37.411 | 7.55 | 33.24 | 46/47 | Ch | Ch | Cyt |
| *AsGLX1-7A* | AVESA.00001b.r1.7Ag0002469.1 | 291 | 32.521 | 5.23 | 23.66 | 45/37 | Cyt | Cyt, Cysk, Ch, Nu | Cyt |
| *AsGLX1-7C1* | AVESA.00001b.r1.7Cg0001974.1 | 291 | 32.564 | 5.32 | 25.13 | 44/37 | Cyt | Cyt, Cysk, Ch, Nu | Cyt |
| *AsGLX1-7C2* | AVESA.00001b.r1.7Cg0001146.1 | 140 | 15.219 | 5.74 | 51.76 | 16/13 | Cyt | Ch, Cyt, Nu | Cyt |
| *AsGLX1-7D* | AVESA.00001b.r1.7Dg0001470.1 | 291 | 32.491 | 5.23 | 23.66 | 45/37 | Cyt | Cyt, Cysk, Ch, Nu | Cyt |
| *AsGLX2-1A* | AVESA.00001b.r1.1Ag0002306.1 | 693 | 77.3 | 5.93 | 41.49 | 86/73 | Cyt | Cyt, Mt, Ch, Nu | Ch |
| *AsGLX2-2A* | AVESA.00001b.r1.2Ag0001489.1 | 331 | 36.832 | 6.13 | 42.98 | 42/37 | Mt | PM, Ch | Mt, Ch |
| *AsGLX2-2C* | AVESA.00001b.r1.2Cg0002204.1 | 331 | 36.772 | 6.37 | 48.11 | 42/39 | Mt | Mt, Ch | Mt, Ch |
| *AsGLX2-2D* | AVESA.00001b.r1.2Dg0000766.1 | 693 | 77.274 | 5.93 | 41.15 | 86/73 | Cyt | Cyt, Mt, Ch, Nu | Ch |
| *AsGLX2-3A* | AVESA.00001b.r1.3Ag0001804.1 | 546 | 59.107 | 8.55 | 42.84 | 48/53 | PM | PM, Ch, Mt | Mt |
| *AsGLX2-3C* | AVESA.00001b.r1.3Cg0001974.1 | 297 | 32.495 | 7.69 | 32.54 | 33/34 | Mt | Mt, Ch | Mt |
| *AsGLX2-3D* | AVESA.00001b.r1.3Dg0001352.1 | 526 | 57.008 | 9.12 | 41.39 | 42/54 | PM | PM, Ch | Mt |
| *AsGLX2-4A* | AVESA.00001b.r1.4Ag0002128.1 | 692 | 77.137 | 5.93 | 42.14 | 86/72 | Cyt | Cyt, Mt, Ch, Nu | Ch |
| *AsGLX2-4D* | AVESA.00001b.r1.4Dg0002447.1 | 1300 | 143.432 | 6.11 | 52.02 | 143/127 | PM | Cyt, Mt, Ch, Nu | Ch |
| *AsGLX2-5A* | AVESA.00001b.r1.5Ag0002862.1 | 311 | 34.443 | 6.67 | 38.35 | 32/31 | Mt | Mt, Ch | Mt |
| *AsGLX2-5C* | AVESA.00001b.r1.5Cg0002496.1 | 311 | 34.403 | 6.67 | 38.63 | 32/30 | Mt | Mt, Ch | Mt |
| *AsGLX2-5D* | AVESA.00001b.r1.5Dg0002414.1 | 311 | 34.443 | 6.67 | 38.35 | 32/31 | Mt | Mt, Ch | Mt |
| *AsGLX2-6C* | AVESA.00001b.r1.6Cg0001537.1 | 693 | 77.314 | 5.93 | 40.61 | 86/73 | Cyt | Cyt, Mt, Ch, Nu | Ch |
| *AsGLX2-7C* | AVESA.00001b.r1.7Cg0000057.1 | 693 | 77.066 | 5.87 | 42.61 | 85/71 | Cyt | Cyt, Mt, Ch, Nu | Ch |
| *AsDJ-1-3A1* | AVESA.00001b.r1.3Ag0000720.1 | 402 | 41.751 | 4.97 | 28.24 | 47/34 | Cyt, Ch | Cyt, Ch, Po | ╱ |
| *AsDJ-1-3A2* | AVESA.00001b.r1.3Ag0000719.1 | 690 | 72.8 | 7.97 | 41.3 | 76/78 | Mt, Ch | Mt, Ch | Ch. Nu |
| *AsDJ-1-3C1* | AVESA.00001b.r1.3Cg0000642.1 | 594 | 62.085 | 5.67 | 56.96 | 56/47 | PM, Ch | Mt, Ch | Nu |
| *AsDJ-1-3C2* | AVESA.00001b.r1.3Cg0000640.1 | 398 | 41.957 | 6.63 | 38.71 | 46/45 | Ch | Mt, Ch | Ch |
| *AsDJ-1-3D1* | AVESA.00001b.r1.3Dg0000176.1 | 398 | 41.235 | 4.9 | 32.5 | 46/32 | Cyt | Cyt, Ch, Po | Ch |
| *AsDJ-1-3D2* | AVESA.00001b.r1.3Dg0000175.1 | 723 | 76.399 | 7.03 | 43.22 | 82/81 | Mt, Ch | Mt, Ch | Ch. Nu |
| *AsDJ-1-4C* | AVESA.00001b.r1.4Cg0001622.1 | 395 | 42.092 | 5.66 | 35.51 | 45/41 | Ch, Cyt | Ch, Cyt, Po | ╱ |
| *AsDJ-1-5C* | AVESA.00001b.r1.5Cg0000441.1 | 450 | 48.264 | 8.02 | 47.91 | 46/47 | Mt, Ch | Mt, Ch | Ch |
| *AsDJ-1-5D* | AVESA.00001b.r1.5Dg0003250.1 | 398 | 42.725 | 5.29 | 41.39 | 47/33 | Cyt | Ch, Cyt | ╱ |
| *AsDJ-1-6A* | AVESA.00001b.r1.6Ag0003093.1 | 659 | 72.519 | 5.84 | 41.12 | 79/66 | Cyt | Mt, Ch | Ch |
| *AsDJ-1-6D* | AVESA.00001b.r1.6Dg0000002.1 | 649 | 70.966 | 9.26 | 52.43 | 69/89 | Cyt | Mt, Ch | Ch |
| *AsDJ-1-7A1* | AVESA.00001b.r1.7Ag0002809.1 | 395 | 41.873 | 5.4 | 32.85 | 46/40 | Cyt | Ch, Cyt, Po | ╱ |
| *AsDJ-1-7A2* | AVESA.00001b.r1.7Ag0001850.1 | 640 | 68.279 | 6.35 | 48.11 | 73/69 | Mt | Cyt, Ch, Nu | Ch |
| *AsDJ-1-7D1* | AVESA.00001b.r1.7Dg0001849.1 | 435 | 46.205 | 6.4 | 42.25 | 46/45 | Ch | Mt, Ch | Ch |
| *AsDJ-1-7D2* | AVESA.00001b.r1.7Dg0000848.1 | 546 | 58.428 | 8.97 | 49.2 | 48/57 | Ch | Ch | Ch |

Note: AA amino acid number, MW molecular weight, pI theoretical isoelectric point, II instability index, NCR negatively charged residues, PCR positively charged residues, Ch chloroplast, Cyt cytosol, Mt mitochondria, Nu nucleus, PM plasma membrane, Po peroxisome, Cysk cytoskeleton.
