# Supplementary material for: Genome-wide analysis and expression profiling of glyoxalase gene families in oat (Avena sativa) indicate their responses to abiotic stress during seed germination
Source: Front Plant Sci. 2023 Jun 15;14:1215084. doi: 10.3389/fpls.2023.1215084 (PMC10308377; doi:10.3389/fpls.2023.1215084)
Supplement: Supplementary file 3 [file Table_3.docx]

**Supplementary Table 3.** Statistics of *cis*-regulatory elements in the promoter region of glyoxalase genes

| *Cis*-regulatory elements | Numbers of elements | | | Proportion of elements | | | Proportion of elements in hormone-responsive elements | | |
| --- | --- | --- | --- | --- | --- | --- | --- | --- | --- |
|  | *AsGLX1* | *AsGLX2* | *AsGLX3* | *AsGLX1* | *AsGLX2* | *AsGLX3* | *AsGLX1* | *AsGLX2* | *AsGLX3* |
| Abscisic acid responsiveness | 177 | 50 | 55 | 0.23 | 0.15 | 0.14 | 0.50 | 0.33 | 0.26 |
| MeJA-responsiveness | 126 | 70 | 116 | 0.17 | 0.21 | 0.29 | 0.35 | 0.46 | 0.54 |
| Auxin responsiveness | 28 | 18 | 16 | 0.04 | 0.05 | 0.04 | 0.08 | 0.12 | 0.08 |
| Gibberellin-responsiveness | 19 | 11 | 21 | 0.03 | 0.03 | 0.05 | 0.05 | 0.07 | 0.10 |
| Salicylic acid responsiveness | 7 | 3 | 5 | 0.01 | 0.01 | 0.01 | 0.02 | 0.02 | 0.02 |
| Low-temperature responsiveness | 24 | 9 | 15 | 0.03 | 0.03 | 0.04 | - | - | - |
| Defense and stress responsiveness | 8 | 4 | 2 | 0.01 | 0.01 | 0.00 | - | - | - |
| Anaerobic induction | 46 | 32 | 26 | 0.06 | 0.10 | 0.06 | - | - | - |
| Light responsiveness | 241 | 78 | 93 | 0.32 | 0.23 | 0.23 | - | - | - |
| Seed-specific regulation | 4 | 3 | 3 | 0.01 | 0.01 | 0.01 | - | - | - |
| Zein metabolism regulation | 10 | 2 | 11 | 0.01 | 0.01 | 0.03 | - | - | - |
| Endosperm expression | 3 | 2 | 9 | 0.00 | 0.01 | 0.02 | - | - | - |
| Meristem expression | 28 | 15 | 8 | 0.04 | 0.05 | 0.02 | - | - | - |
| MYB binding site | 39 | 35 | 22 | 0.05 | 0.11 | 0.05 | - | - | - |
